# A scoping review of alopecia areata and its relationship to COVID-19 vaccinations

**DOI:** 10.1007/s00403-024-03583-z

**Published:** 2024-12-12

**Authors:** Hailey Konisky, Solbie Choi, Aditi Valada, Tyler M. Andriano, Kseniya Kobets

**Affiliations:** Albert Einstein College of Medicine, Montefiore Medical Center, 1300 Morris Park Avenue, Bronx, NY 10461 USA

**Keywords:** Alopecia areata, Hair loss, Vaccine, COVID-19 vaccine, SARS-CoV-2 vaccine, Alopecia universalis, Alopecia totalis

## Abstract

In response to the COVID-19 pandemic several vaccines were produced, including novel mRNA and viral vector-based vaccines. Though COVID-19 had its own associated dermatological sequelae, the vaccines were associated with a new set of cutaneous side effects, including hypersensitivity reactions, vasculitis, and autoimmune-mediated reactions. Notably, alopecia areata (AA) was reported in several patients closely following a COVID-19 vaccine, especially in those with a personal or family history of AA. A PubMed and Google Scholar search was conducted in July 2024 which resulted in 26 case reports/case series, 1 prospective study, and 3 cross-sectional retrospective chart reviews. Based on our holistic literature review, there is no evidence to support an increased association between COVID-19 vaccination and AA. Despite recent literature highlighting the incidence of *de novo* and recurrent AA after COVID-19 vaccines, several large retrospective analyses have shown that the overall incidence of AA in vaccinated individuals does not differ from that of historical controls. The potential for *de novo* AA after COVID-19 vaccine is low and the benefit of being vaccinated far outweighed the risks, especially within the first few years of COVID-19 vaccine rollout. While the decision to get vaccinated is a personal choice, the threat of developing AA secondary to vaccination should not be a deterrent.

## Introduction

In response to the COVID-19 pandemic several vaccines were produced, including novel mRNA and viral vector-based vaccines. Though COVID-19 had its own associated dermatological sequelae, the vaccines were associated with a new set of cutaneous side effects, including hypersensitivity reactions, vasculitis, and autoimmune-mediated reactions [24]. Notably, alopecia areata (AA) was reported in several patients closely following a COVID-19 vaccine, especially in those with a personal or family history of AA. Other vaccines have also been implicated in precipitating AA, including herpes zoster, hepatitis B, and HPV [1, 10]. As of June 8, 2024, the CDC Vaccine Adverse Event Reporting System (VAERS) logged 467 reports of AA after any brand of COVID-19 vaccine. A recent meta-analysis of 51 patients noted that 53% of patients experienced *de novo* AA after COVID-19 vaccination [33]. The purpose of this review is to provide a comprehensive overview of studies describing COVID-19 vaccine-associated AA to further enrich our understanding of this phenomenon.

## Methods

A PubMed and Google Scholar search was conducted in July 2024 using combinations of the terms “alopecia areata” AND “vaccine”, “alopecia areata” AND “SARS-CoV-2 vaccine”, and “hair loss” AND “COVID-19 vaccine”. Papers were excluded if they were not in English, were review papers, discussed hair loss due to COVID-19 disease or hair loss other than alopecia areata, or discussed a vaccine other than COVID-19. This resulted in 26 case reports/case series, 1 prospective study, and 3 cross-sectional retrospective chart reviews.

## Results

### Cross-sectional studies

A 2022 nationwide assessment in South Korea compared over 3 million vaccinated individuals to matched controls. Overall, there was not an increased incidence of any autoimmune disease post- COVID-19 vaccination. They noted that the incidence of AA, alopecia totalis, and cicatricial alopecia did not differ significantly between vaccinated individuals and historical controls. The authors postulate that the association of increased autoimmune disease after the COVID-19 vaccine is a result of intensive observation of patients and physicians after administration of a new vaccine [19].

A retrospective review conducted in Italy reviewed patients at a hair loss clinic from 2021 to 2022. They found that 16% of patients at this clinic had AA, with no statistical difference from pre-COVID historical controls (*p *= 0.244). Twenty-four patients were identified as having AA after COVID-19 vaccination. Pfizer BNT162b2 was the most common vaccine amongst these individuals (62%), and onset of hair loss was on average 5.2 weeks from vaccination of any type. The average age was 39.1, and there was a strong female predominance (83%). In 22 of these patients, the hair loss was a relapse of prior AA. Half of the patients had a history of other autoimmune diseases. This study overall highlights that while there was not an increased incidence in AA because of the COVID-19 vaccine, female patients with a history of AA or other autoimmune conditions are at a higher risk of developing relapse of AA within 16 weeks of vaccination [29].

Another retrospective cohort study from California, USA, analyzed 73 cases of AA diagnosed in their clinic between December 2020 and February 2023 and compared to matched controls. Thirty-seven of these patients received 1–2 doses of the COVID-19 vaccine prior to AA onset. In contrast to other studies, the authors found that the odds of developing AA after vaccination was decreased (OR 0.58, 95% CI = 0.35–0.94). The risk of AA was also found to be significantly lower in females. The authors acknowledge limitations to this study, but postulate that this finding could be due to modulation of regulatory T-cells after vaccination which mitigates autoimmune activity, or due to reduced anxiety and stress after being vaccinated during a global pandemic [8].

### Prospective study

A prospective study explored the causality, immune mechanism, and clinical manifestations of immune-mediated hair loss associated with COVID-19 vaccination in 27 patients with a new-onset AA following vaccination compared to 106 vaccinated controls. The mean age of the AA group was 38.2 ± 15.8 years versus 43.7 ± 15.9 years in controls. Of those with AA, 40.7% received the mRNA-1273 vaccine, 29.6% received the BNT162b2 vaccine, 25.9% received the AZD1222 vaccine, and 3.7% received the MVC-COV1901 vaccine. Sixteen patients (59.3%) experienced symptoms after the 2nd dose of the vaccine, 5 patients after the 3rd/4th dose (18.5%), and 6 patients after the 1st dose (22.2%). The mean time from vaccination to AA onset was 27.6 ± 19.7 days (range: 2–65 days), with time to symptom onset inversely correlating with the number of doses.

Pertinent history of autoimmune diseases including atopic dermatitis (7.4%, *n* = 2), urticaria/chronic urticaria (7.4%, *n* = 2), pompholyx (3.7%), ankylosing spondylitis (3.7%), and androgenetic alopecia (3.7%) were noted. There were no significant differences between groups regarding autoimmune history. Laboratory data revealed significantly increased antinuclear antibody (ANA) level (OR = 6.4, 95% CI = 1.6–25.8), total IgE level (OR = 8.0, 95% CI = 2.8–23.1), and eosinophil count (> 5%) (OR = 3.8, 95% CI = 1.2–12.1) in the AA groups versus controls. There were no cytokines, chemokines, or inflammatory proteins levels that correlated with the severity of AA.

Eleven (40.7%) patients in the AA group were categorized as severe using the SALT scale, and 16 (59.3%) were categorized as having mild to moderate AA. Twenty-five of the 27 AA patients were on multiple agent therapy. Topical corticosteroids were the most common form of treatment (*n* = 24), and over 81.5% were on at least one systemic immunosuppressive agent. JAK inhibitors (tofacitinib, baricitinib) were administered to 6 patients with severe and recalcitrant AA. Of the AA group, 14 patients reached complete remission after > 5 months of follow-up. The average treatment duration of this group was 153.2 ± 57.3 days [31]. This study demonstrates that individuals with elevated blood markers of autoimmunity are more likely to have new or recurring AA after COVID-19 vaccination.

### Selected case reports

Case reports described patients developing AA after various vaccine formulations: Pfizer (*n* = 40), Moderna (*n* = 14), AstraZeneca (*n* = 14), and Sinopharm (*n* = 5). There was a predominance of females affected (*n* = 51/73), which is typical for autoimmune conditions [3]. Comorbid AA or thyroiditis was often observed, in addition to a personal or family history of autoimmunity. There was a recurrence or worsening of prior AA within 21 patients who reported a personal history of AA. Onset of AA after the vaccine varied from days to months. Treatments such as clobetasol, 5% minoxidil, intralesional triamcinolone (ILTAC), tofacitinib citrate, triamcinolone, oral prednisolone, hydroxychloroquine, dexamethasone, corticosteroids, and JAK inhibitors were attempted, but did not always show effective results. A total of 73 individuals with post-vaccination AA, either *de novo* or recurrent, were described in case series or reports (Table 1). Herein, we highlight a select few.

## Recurrence of alopecia areata

Abdalla et al. presented a 63-year-old female with a history of hypothyroidism, prediabetes, thalassemia trait, and allergic rhinitis. She experienced a recurrence of AA after 32 years of remission 2 weeks after receiving the first dose of the BNT162b2 vaccine, initially presenting with patchy hair loss which progressed to AA universalis within 6 weeks of the first dose [1].

Scollan et al. presented a 28-year-old woman with a history of AA and Hashimoto thyroiditis who reported loss of all scalp and body hair within 1 week after her 2nd dose of the BNT162b2 series. Treatment with intralesional triamcinolone (ILTAC) and platelet-rich plasma (PRP) therapy provided little improvement. The patient was subsequently prescribed tofacitinib citrate 10 mg twice a day [26].

Rossi et al. presented a 29-year-old female with a medical history of patchy AA, previously treated with topical and systemic steroids with complete regrowth. Two weeks following her first dose of the AZD1222 vaccine, she presented with sudden generalized hair loss on the scalp with a partial loss of eyebrows and eyelashes. Trichoscopy revealed multiple black dots and broken hairs. The patient was subsequently diagnosed with acute AA and began a treatment regimen of topical steroids with a follow-up scheduled in 4 weeks [25].

## *De novo* alopecia areata

May Lee et al. presented an 80-year-old male with no relevant past medical history who experienced rapid beard hair loss on his left cheek and upper lip and scalp hair loss 7 days after his first dose of the BNT162b2 vaccine. AA was diagnosed via a positive pull test and trichoscopy examination showing cadaveric and exclamation point hairs. His AA worsened after the second dose of the vaccine [23].

Hernandez Arroyo et al. presented a 59-year-old male patient with no family or personal history of autoimmune disease and a remote past medical history of COVID-19 who presented with excessive hair loss 17 days following the booster of the AZD1222 vaccine. He was diagnosed with AA universalis. He was treated with mesotherapy and pulses of dexamethasone and clobetasol propionate 0.5% topically with 15% improvement in regrowth [16].

Iwata et al. presented a Japanese female patient in her 40s with no relevant history of AA who developed loss of hair on her scalp, eyebrows, eyelashes, axilla, pubis, arms, and legs one week after her first dose of the mRNA-1273 vaccine. She was treated with prednisone, excimer lamp therapy, oral cepharanthine, and monoammonium glycyrrhizinate but showed minimal improvement [18].

## Discussion

Based on our holistic literature review, there is no evidence to support an increased association between COVID-19 vaccination and AA. Recent meta-analysis causality assessments noted that only 20% of cases were classified as “consistent with causal association to immunization” versus 53% that were “coincidentally associated” [33]. The BNT162b2 (Pfizer) vaccine was the most implicated in the literature; however, this may be due to lead-time bias since it was the first FDA-approved COVID-19 vaccine.

AA after infection or vaccination against COVID-19 is thought to be due to molecular mimicry, since the spike protein of the virus is closely related to some human proteins [24]. AA is a multifactorial autoimmune disease mediated by T-cells that attack the hair follicle causing loss of immune privilege [31]. One prospective study identified that vaccine ingredients such as PEG2000 and polysorbate 80 triggered a similar immune response as exposure to the SARS-CoV-2 spike protein. Additionally, patients with vaccine-induced AA had significantly higher levels of cytotoxic proteins from CD8 + T-cells that may cause cell apoptosis in the hair follicle and trigger the autoimmune response [31]. It is postulated that the vaccine may also quickly increase interferon, which upregulates IL-12 and IL-23 causing inflammation [10].

Despite recent literature highlighting the incidence of *de novo* and recurrent AA after COVID-19 vaccines, several large retrospective analyses have shown that the overall incidence of AA in vaccinated individuals does not differ from that of historical controls. Those authors concluded that the potential for *de novo* AA after COVID-19 vaccine is low and that the benefit of being vaccinated far outweighed the risks, especially within the first few years of COVID-19 vaccine rollout. While the decision to get vaccinated is a personal choice, the threat of developing AA secondary to vaccination should not be a deterrent.

## Conclusions

Overall, studies have shown that vaccination against COVID-19 does not increase the incidence of AA. However, variables that may predispose individuals to AA catalyzed by this vaccine include female sex, personal history of AA, and personal or family history of autoimmunity. The risk of AA secondary to COVID-19 vaccination remains low when compared to the individual and public health benefits. Future research on AA and its relationship to vaccines, including the COVID-19 vaccine, are warranted to further elucidate the mechanism in which this phenomenon occurs and how to best counsel patients.

**Table 1:** Summary of cases described in the literature from March 2020–July 2024

PMID	Patients	Type of SARS-CoV-2 vaccine	History of autoimmunity	Time from vaccine to hair loss	Extent of AA	Significant family history	Clinical presentation	Treatment/ Outcome
Case reports								
35975094 [1]	*n* = 1 65 F	1st dose (BNT162b2)	Hashimoto’s Thyroiditis Allergic rhinitis AA 32 years ago	2 weeks after 1st dose	PAA → AAU	N/A	Complete loss of scalp, facial, and body hair after 6 weeks	N/A
36239007 [21]	*n* = 1 7 F	2nd dose (BNT162b2)	AA	20 days after 2nd dose	PAA	N/A	Nonscarring alopecia patch on occipital region with foci of hair regrowth	N/A
35107173 [23]	*n* = 1 80 M	1st dose (BNT162b2)	N/A	7 days after 1st dose	AAU→ AAT	N/A	Beard hair loss on left cheek Diffuse hair loss in scalp Complete hair loss in scalp 2 month follow up showed AAT	Failed: foam clobetasol Failed: a topical immunotherapy with squaric acid dibutylester combined with topical 5% minoxidil.
35223675 [12]	*n* = 1 31 M	2nd dose (BNT162b2)	N/A	1 day after 2nd dose	AAU	N/A	Multiple circular patches of alopecia on occipital, bilateral parieto-temporal, front area with beard	N/A
34559937 [9]	*n* = 1 32 F	(AZD1222)	Mild attack of AA but stable for 6 years previous COVID-19 1 year prior	Few days after dose	PAA	N/A	Sharply demarcated patchy hair loss on scalp without scarring or scaring	N/A
35642187 [17]	*n* = 1 51 F	2nd dose (AZD1222)	N/A	3 days	AAU	N/A	Intense pruritus of scalp with subsequent complete loss of all body hair in 3 weeks	Clobetasol propionate ointment -Intralesional triamcinolone acetonide 10 mg/mL
35770484 [13]	*n* = 1 27 F	Booster (BNT162b2)	Systemic lupus erythematosus after AZD1222 one year prior	15 days after dose	AA	N/A	diffuse alopecia, bx proven AA	N/A (Only treatment of SLE listed − 80 mg/day oral prednisolone and hydroxychloroquine 400 mg/day.)
35379574 [28]	*n* = 1 42 M	1st dose (AZD1222)	N/A	3 weeks after 1st dose	PAA	N/A	Well demarcated patches of hair loss (1–10 cm) on scalp, without scarring or scaling Largest patch at vertex	ILTAC (10 mg/dL)
37441583 [18]	*n* = 1 40s F	(mRNA-1273)	N/A	1 week after 1st dose	AAT, AAU	N/A	Hair loss on head, eyebrows, eyelashes, axilla, pubis, arms, legs	Failed: Oral prednisone, oral cepharanthin, monoammonium glycyrrhizinate Little improvement: Excimer lamp treatment
36856386 [32]	*n* = 1 20 F	Booster (Sinopharm)	N/A	2 weeks after booster dose	PAA	N/A	Several patches of non-scarring alopecia on her scalp SALT score: 30	Topical and oral steroids
No PMID Matsuda et al. 2023 [22]	*n* = 1 37 F	(BNT162b2)	N/A	22 days after 1st dose	PAA→ AAT	N/A	Coin-sized patches of hair loss on scalp, progressing to greater area on scalp	Signs of hair growth on day 120 with topical betamethasone butyrate propionate lotion Almost full recovery on day 310
35571458 [20]	*n* = 1 61 F	2nd dose (BNT162b2)	N/A	1 week	PAA	N/A	4 areas of scalp patchy hair loss, 2 punch biopsies revealed follicular miniaturization, catagen/telogen shift, peribulbar/lymphocytic inflammatory infiltrate	0.05% topical fluocinonide solution, topical minoxidil for 1 month, subsequent 0.1% tacrolimus ointment and 5% minoxidil solution for additional 5 months Intralesional acetonide 2.5 mg/cc administered once a month for 3 consecutive months Improvement observed 1 month after treatment, full regrowth in four areas observed at 4 month follow up No relapse in hair loss observed after Pfizer booster vaccine or 8 month follow up
37192849 [30]	*n* = 1 34 F	2nd dose (AZD1222)	N/A	4 weeks after 2nd dose	AA	N/A	Scalp hair loss over entire scalp, hair pull test revealed massive hair loss after slight pull	Ineffective: Oral prednisolone 25–40 mg daily for approximately 1 month Subsequent: double-spin platelet-rich plasma (PRP) therapy from 80mL of whole blood, second dose of PRP (160mL whole blood) 6 weeks later; Hair regrowth was observed and hair loss stabilized after two doses of PRP treatment; Complete recovery after 6 courses of PRP treatment
36942081 [2]	*n* = 1 44 M	2 doses (mRNA-1273)	N/A	2 weeks after 1st dose	AA	N/A	SARS-CoV2 infection followed by vaccination 3 months later Patches of scalp hair loss following first dose; Diffuse hair loss (scalp, eyelashes, bear hair, eyebrows) following second dose Facial and scalp hair regrowth 2 weeks after second dose followed by subsequent Marie Antoinette syndrome 3 days after regrowth. New-onset arm and lower leg hair loss	Ineffective treatment following first dose: 50 mg PO Prednisone daily for 5 days, betamethasone valerate 0.05% lotion (1 month discontinue due to dermatitis, abscesses)
35757867 [4]	*n* = 1 33 F	2 doses (Sinopharm)	N/A	4 weeks after 2nd dose	AA	N/A	Delineated hair loss patches with no scarring or scaling compromising < 50% of scalp	Intralesional and topical corticosteroids 1-month follow-up to treatment revealed stable lesions and signs of regrowth
Case Series								
35789521 [7]	*n* = 2 1: 29 M 2: 26 F	1: (AZD1222) 2:(BNT162b2)	1: AA 2: AA	1: 1 week after 2nd dose 2: 2 weeks after 2nd dose	1: PAA 2: PAA→ AAU	N/A	1: Balding patches on scalp 2: Initial diffused hair loss on scalp and body, progressing to areas of eyebrows and eyelash	1 Failed: pulse steroid therapy 2 Failed: pulse steroid therapy
36172335 [14]	*n* = 2 1: 23 F 2: 26 F	1: (AZD1222) 2: (AZD1222)	1: N/A 2: AA	1: 1 week after 1st dose 2: 2 weeks after 2nd dose	1: PAA→AAU 2: PAA→ AAT	N/A	1: Myalgia and local hair scalp, progressing to entire loss of hair in scalp and eyebrows after 3 weeks 2: Patchy hair loss in scalp progressing to total hair loss in scalp after 1 month	1: Systemic cortisone (oral prednisone, 300 mg) for three months 2: Systemic cortisone (oral prednisone, 300 mg) for three months
36926281 [15]	*n* = 5 4 F: Mean Age 32.4 1 M: 31y	2 patients: (BNT162b2) 3 patients: (mRNA-1273)	AA (remission 1.8 years ± 1.48)	Mean time after 1st dose: 2 weeks ± 0.7	AAT	N/A	Hair loss was 25% greater than at the beginning	Failed: minoxidil 5% 1 mL bis in die, topical clobetasol, topical growth factors, and intralesional triamcinolone 3:1
34931171 [26]	*n* = 9 Mean Age: 35.9 1:33 F 2:57 F 3: 62 F 4: 28 F 5: 29 F 6: 22 M 7 :15 M 8: 61 M 9: 16 M	1:mRNA-1273 2: BNT162b2 3:mRNA-1273 4: BNT162b2 5: BNT162b2 6: BNT162b2 7: BNT162b2 8: BNT162b2 9: BNT162b2	1: N/A 2: remote history AA 3: remote Hx AA 4: AA, Hashimoto thyroiditis 5: Elevated levels of thyroglobulin antibody and thyroid peroxidase Antibody 6: Elevated thyroid antibody, normal thyroid function Tests 7: NA 8: Joint pain hydroxychloroquine 9: NA	1: 2 months after 2nd dose 2: 4 months after 2nd dose 3: 2 months after 2nd dose 4: 1 week after 2nd dose 5: 1 week after 2nd dose 6: 1 month after 2nd dose 7: Within 1 week after 2nd dose 8: 2 weeks after 1st dose 9: Within 1–2 weeks after 1st dose	1: PAA 2: AAT 3: AAU 4: AAU 5: PAA 6: PAA 7: PAA 8: AAT 9: PAA	1: Brother AA 7: Grandmother with Hashimoto thyroiditis, sister w/ elevated thyroid antibody	1: Large patches of nonscarring alopecia with foci of hair regrowth 2: widespread nonscarring alopecia of scalp with foci of hair regrowth 3: alopecia universalis 4: Alopecia universalis 5: Two patches of nonscarring alopecia of the scalp with areas of regrowth 6: Patches of nonscarring alopecia; 30% hair loss from scalp, 80% hair loss from beard 7: Two patches of nonscarring alopecia of the scalp 8: Alopecia totalis 9: Patches of nonscarring alopecia with 70% loss of scalp hair; sparse eyebrows and eyelashes	1: 4 treatments intralesional triamcinolone (ILTACT), pimecrolimus 1% cream, clobetasol 0.05%foam, subsequent tofacitinib citrate 5 mg 2: Failed: attempted trials tofacitinib 2%/clobetasol 0.05% ointment/clobetasol solution compounded with little improvement Subsequent Tx: tofacitinib citrate 5 mg 3: tofacitinib citrate 10 mg 2x/day, bimatoprost 0.03% eye drops 4: Failed: PRP therapy (little improvement) Subsequent: tofacitinib citrate 10 mg 2x/day 5: NA previous, subsequent ILTAC 6: Failed previous: ILTAC subsequent Tofacitinib citrate 10 mg twice a day 7: ILTAC 8: Pending possible trial of oral tofacitinib citrate 9: Failed: ILTAC Subsequent: Tofacitinib citrate 10 mg twice a day
35182005 [6]	*n* = 3 4: 41 F 5: 24 M 6: 21 F	4: 1st dose BNT162b2 5: 1st dose BNT162b2 6: 1st dose mRNA-1273	5: Previous episodes of AA in pediatric age. 6: Allergic Asthma	4: 1 week after dose 5: 1 week after dose 6: 2 weeks after dose	4: PAA 5: PAA 6: PAA	5: Previous episodes of AA in pediatric age. Sister affected by AGA + AAI	4:Patchy (multiple patches) SALT: 26; Complete regrowth in 3 months 5:AA totalis SALT: 100; Alopecia universalis.	5: Intramuscular triamcinolone 40 mg/dL every 4 weeks for 3 sessions + clobetasol propionate 0.05% under occlusion 5 times/week 6:3 sessions of monthly injection of intralesional triamcinolone 10 g/mL
36851320 [16]	*n* = 3 1: 27 F 2: 51 F 5: 59 M	Booster (3rd dose) (BNT162b2, Sinopharm, and AZD1222)	1: AAU, Polycystic Ovary, COVID-19 2: N/A 3: N/A	1: 8 days after dose 2: 3 days after dose 5: 17 days after dose	1: AAU 2: AAU 5: AAU	2: grandmother with hypothyroidism	1:Alopecia universalis 5:Alopecia universalis	1: Mesotherapy and pulses of dexamethasone and Clobetasol propionate 0.5% topical 5: Mesotherapy and pulses of dexamethasone and Clobetasol propionate 0.5% topical
34741583 [25]	*n* = 3 1: 76 F 2: 59 F 3: 29 F	1: BNT162b2 2: AZD1222 3: AZD1222	1: AA 2: AA, autoimmune thyroiditis 3: AA	1: 2 weeks 2: 3 weeks 3: 2 weeks	1: AA 2: AA 3: AA	N/A	1: Widespread hair loss of scalp 2: Single oval bald patch at vertex 3: Generalized hair loss of scalp	1: topical steroids 2: topical steroids, oral Vitamin D 3: steroids
36146545 [29]	*n* = 24 (20 F, 4 M) Mean Age: 39.1	15 with 2–3 doses BNT162b2 5 with 2 doses mRNA-1273 4 with 2 doses AZD1222	12 with Autoimmune History 9 with Hashimoto thyroiditis 2 with Celiac disease 1 with Connective tissue disease	BNT162b2- 5.7 weeks mRNA-1273- 6.4 weeks AZD1222- 2.2 weeks	PAA- (*n* = 14) AAT (*n* = 6) AAU (*n* = 4)	N/A	N/A	76% responded to treatment with topical and/or systemic treatment.
35716105 [27]	*n* = 2 1–74 M 2–37 M	1- Sinopharm 1st dose 2- Sinopharm 2nd dose	N/A	1: 2 days 2: 6 days	1: PAA 2:PAA→AAT	N/A	N/A	ILK
36035747 [5]	*n* = 3 1–33 F 2–27 M 3–32 M	1–3 doses BNT162b2 2-2 doses BNT162b2 3-2 doses mRNA-1273	N/A	1: 2 weeks after first dose, exacerbated after second dose 2: 2 weeks after second dose 3: 2 weeks after first dose	1: AA 2: AA 3: AA	N/A	1: near complete, widespread loss of scalp hair 3 months following second dose (SALT increase 33) 2: Patchy Scalp Hair Loss 3: Patchy Scalp Hair loss	Pre-vaccination JAKi therapy continued, no changes to systemic therapy
36483229 [11]	*n* = 2 1–42 M 2–18 M	1:3 doses BNT162b2 2: 2 doses BNT162b2	1: AA 2: PAA	1: 4 weeks after second dose, worsened after third dose 2: 20 days after first dose, worsened progressively after second dose	1: AT 2: AT	N/A	1: complete loss of whole scalp hairs 2: pre-existing AA patches being treated, experienced worsening of patches being treated following first dose; after second dose, widespread hair loss on whole scalp, positive pull test	1: High potency steroids 2: High potency steroids

ChAdOx1 nCoV-19 = AZD1222 = AstraZeneca Recombinant Vaccine

mRNA-1273 = Spikevax^®^= Moderna mRNA

BNT162b2 = Comirnaty^®^= Pfizer BioNTech mRNA

MVC-COV1901 = Medigen Recombinant Vaccine

Sinopharm = CoronaVac^®^= inactivated virus

M Male, F Female, PAA Patchy alopecia areata, AAT Alopecia areata totalis, AAU Alopecia areata universalis, AA Alopecia areata, N/A Not applicable, SALT The severity of alopecia tool

## Data Availability

No datasets were generated or analysed during the current study.
